# Therapeutic Prospects of mRNA-Based Gene Therapy for Glioblastoma

**DOI:** 10.3389/fonc.2019.01208

**Published:** 2019-11-08

**Authors:** Xiangjun Tang, Shenqi Zhang, Rui Fu, Li Zhang, Kuanming Huang, Hao Peng, Longjun Dai, Qianxue Chen

**Affiliations:** ^1^Department of Neurosurgery, Renmin Hospital of Wuhan University, Wuhan, China; ^2^Department of Neurosurgery, Taihe Hospital, Hubei University of Medicine, Shiyan, China; ^3^Department of Neurosurgery, Affiliated Hospital of Xi'an Jiaotong, University Health Science Center, Xi'an, China; ^4^Department of Surgery, University of British Columbia, Vancouver, BC, Canada

**Keywords:** mRNA, gene therapy, glioblastoma, patient-tailored, targeting molecules

## Abstract

The treatment of glioblastoma has been a big challenge for decades in the oncological field mainly owing to its unique biological characteristics, such as high heterogeneity, diffusing invasiveness, and capacity to resist conventional therapies. The mRNA-based therapeutic modality holds many superior features, including easy manipulation, rapid and transient expression, and adaptive convertibility without mutagenesis, which are suitable for dealing with glioblastoma's complexity and variability. Synthetic anticancer mRNAs carried by various vehicles act as the ultimate attackers of the tumor across biological barriers. In this modality, specifically targeted glioblastoma treatment can be guaranteed by adding targeting molecules at certain levels. The choice of mRNA-bearing vehicle and administration method is a fully patient-tailored selection. This review covers the advantages and possible limitations of mRNA-based gene therapy, the *in vitro* synthesis of mRNA, the feasible methods for synthetic mRNA delivery and clinical therapeutic prospects of mRNA-based gene therapy for glioblastoma.

## Background

Glioblastoma multiforme (GBM) is the most common and aggressive primary brain tumor with inferior prognosis. GBM is thought to arise from the neuroglial stem or progenitor cells and has been defined by WHO as a grade IV glioma (1, 2). It is recurrent in almost all patients (3). GBM affects 3–4 people out of every 100,000 per year, with a sustained and highly significant incidence rise across all ages (4, 5). The outcomes for treating GBM retain gloomy, though surgical techniques and adjuvant therapies have progressively developed for decades. GBM remains a virtually incurable disease, resulting in a death rate of greater than 95% within 5 years of diagnosis (6). The main reasons that GBM is challenging to treat relate both the restriction of surgical resection and the resistance to irradiation and chemotherapy (7). Not only the drugs were prevented to enter into gliomas' cells by blood-brain barrier (BBB) and brain-tumor barrier (BTB) in the brain, but also the complexity of tumor composition and diffusing invasiveness have hindered the better effective treatment for over three decades (8, 9). There is an urgent need for advancement in treatment strategies to improve outcomes for GBM patients. Since Wolff et al. pioneered the concept of nucleic acid based therapy, reporting functional protein expression in target organs after the direct injection of plasmid DNA or mRNA (10), gene therapy has held a great potential to provide a viable alternative to conventional treatments toward effectively overcoming cancer progression in GBM (11).

Till now, four main types of vectors have been widely used in gene-related therapy, including plasmids, viral vectors, cosmids, and artificial chromosomes. Viral vectors used for gene therapy are associated with severe safety issues (12, 13). Meanwhile, low transfection efficiency and the potential to induce mutations limited the development of non-viral DNA vectors (14). Recent research reported that novel stabilized mRNA constructs have become more attractive alternatives to the most commonly used DNA-based plasmid (pDNA) (15, 16), mainly due to their ease of manipulation and safety in clinical applications. This review discusses the advantages and possible limitations of mRNA-based gene therapy, the feasible methods for mRNA-based gene delivery and clinical therapeutic prospects of mRNA-based gene therapy for glioblastoma.

## The Beneficial Aspects of mRNA-based Gene Transfer

Synthetic mRNA has emerged as an efficient gene transfection tool, and a wide range of therapeutic applications have been developed (17). Prolonged intracellular persistence of mRNA is a basic prerequisite for synthetic mRNA to be effectively used in gene therapy. mRNA-based gene have significantly enhanced translational efficience of foreign mRNA in host cells, mainly owe to the discovery of 5′mRNA anti-reverse cap analogs (ARCA), the insertion of additional untranslated regions, and poly(A) tails (15, 18–20). To summarize, compared with pDNA delivery in gene therapy, mRNA-based gene treatment has more significant virtues: (i) pDNA is translated into the nucleus and mRNA is translated in the cytoplasm directly. The mRNA transfection is efficient even in quiescent cells, which is obviously different from pDNA transfection; (ii) the risk of insertional mutagenesis can be ignored by the nature of RNA. Hence, mRNA has a significant security compared to DNA in gene therapy for clinical applications; (iii) the immunogenic reaction of toll-like receptor-activated mRNA is weaker than the unmethylated CpG motifs of DNA recognized by TLR9; (iv) the mRNA transfection into host cells can be much easier because its construct is far smaller than pDNA. Furthermore, mRNA gene therapy circumvents the need for selecting a specific promoter, and thus the transfection process is relatively efficient and facile; (v) protein translation takes place almost immediately after mRNA transfection because of it's functionality in the cytoplasm without the need to enter into the nucleus (15, 21–24). Mainly because of the unstable structure and ubiquitous presence of RNase, the biggest concern about mRNA-based gene vehicle has been its stability and durability during its application. Table 1 lists the comparisons between mRNA- and DNA-based gene carriers as regards to practical applications.

**Table 1:** Comparisons between mRNA- and DNA-based gene carriers.

**Immunogenicity**	**Low**	**High**	**(104)**
Target cell type	Dividing and non-dividing cells	Dividing cells	(17)
Potential mutation	None	Possible	(25)
Cellular delivery	Much easier	More difficult	
Therapeutic action	Rapid (hours)	Delayed (3–5 days)	(25)
Production cost	High	Low	(26)
“Advanced therapy medicinal products” regulation required	No	Yes	(25)

## *In vitro* Synthesization of Target Gene-bearing mRNA

**Figure 1:**
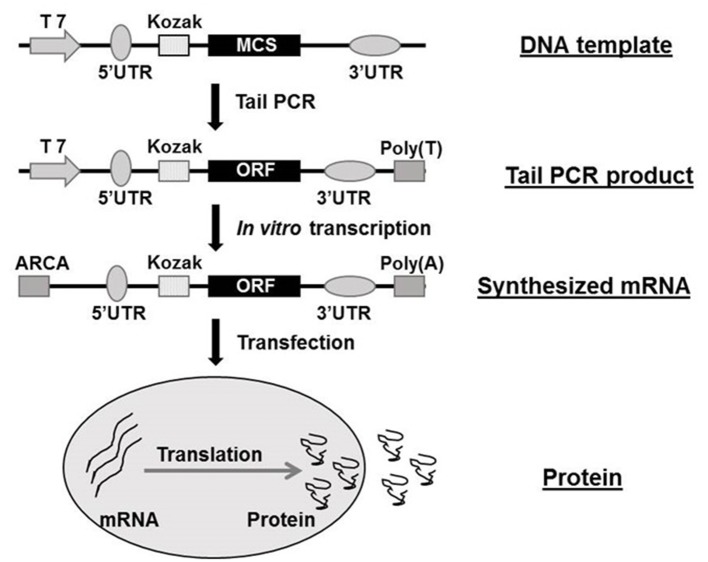
Flow chat of mRNA synthesization *in vitro*. ARCA, anti-reverse cap analog; MCS, multiple cloning site; ORF, open reading frame; PCR, polymerase chain reaction; UTR, untranslated region. The flow chat was reproduced from Guo et al. (16).

The structure of synthetic mRNA is the same as the structure of natural mRNA, containing a cap, 5′ and 3′ UTRs and a poly(A)-tail and the encoded gene of interest. Regardless of the application, the vital factor is bioavailability of the synthetic mRNA. In recent years, the cap structure of the eukaryotic mRNAs naturally occurring at the 5′end has been studied on the therapeutic use of mRNAs (22, 27, 28). The cap is involved in mRNA's maturation, nuclear export, initiation of translation, and their turnover through interacting with highly specialized cap-binding proteins (29, 30). Due to the existence of NTPs and a regular cap such as m7GpppGNpN, polymerase-mediated transcripts are highly capped in a reverse orientation (i.e., Gpppm7GpNpN) up to one-third to one-half of total transcripts (28, 31). Such reverse-capped transcripts significantly reduce the translational efficiency of mRNA. However, 3′-O-methyl, 3′-H, or 2′-O-methyl modified anti-reverse cap analogs (ARCAs) of the m7Guo can achieve 100% correct orientation, thereby resulting in higher translational efficiency of synthetic mRNAs (18, 32–34). It can also improve resistance to enzymatic degradation (35). ARCA is now widely used in *in vitro* synthesization of mRNA. 3′ UTR is another key regulator of intracellular kinetics of an mRNA molecule (36). The length of the 3′ UTR is a critical factor since the longer of the mRNAs 3′ UTRs the shorter of the half-life, meanwhile mRNAs with shorter 3′ UTRs are less efficiently translated (37, 38). Human globin 3′ UTRs are now being commonly used in mRNA synthesization, mainly based on the distinctive feature of human erythrocytes (17). Practically, the human 5′ UTR with Kozak sequence, standardized 3′ UTR sequence and ARCA cap analog are all commercially available. The presence and length of the 3′-poly(A)-tail in mRNA also have great importance for efficient translation and stability (39). Different administration route may result in diverse average half-life of protein production from transfected modified mRNA, it ranges from 50 h *in vitro* to 7–30 h *in vivo* (40). The majority of mRNA decay are began with deadenylation of the poly(A)-tail total to ~10 nucleotides (41, 42), so a poly (T_120_) sequence was always introduced in the Tail PCR process in our previous studies (16, 20). The Figure 1 shows the flow chart of mRNA synthesis *in vitro*.

## Delivery of Synthesized mRNA

Synthetic mRNAs have to be located in the cytoplasm of targeted cells to be translated into corresponding proteins using the host cell's machinery. As Kowalski et al. indicated, the delivery of exogenous synthetic mRNA is quite complex and requiring overcome numerous obstacles in extra- and intra-cellular to reach the cytoplasm exogenous synthetic mRNA delivery process is quite multifaceted and faces multiple extra- and intra-cellular barriers to reach the cytoplasm (43). Under most circumstances, the cell membrane is a major and formidable barrier for synthetic mRNA's intracellular delivery. The characteristics of mRNA and lipid bilayer components of the cell membrane are essential factors when we design an ideal method to deliver synthetic mRNAs to targeted cells. In general, the delivery of mRNA in intracellular is not as easy as small oligonucleotides, in part because of its larger size (300–5,000 kDa, up to 15 kb) and poly-anionic feature, and it needs encapsulation into nanoparticle (43–45). The cell membrane is primarily made up of a lipid bilayer composed of zwitterionic and negatively charged phospholipids (43). The polar heads of the phospholipids point toward the aqueous environment and the hydrophobic tails form a hydrophobic core (43, 46). The ion channels and ion pumps mounted in the lipid bilayer maintain a negative potential (−40 to −80 mV) across the cell membrane, forming an electro-barrier for highly negatively charged mRNA molecules (47). Thus, appropriate supplementary measures are required to facilitate synthetic mRNA crossing the cell membrane. It is amazingly stable and sustained long time if the mRNA transferred into the cytoplasm (22). Since the relatively small size and single chain structure of mRNA, majority of the available delivery tools were shown to work better with mRNA which were well used for studying in plasmid DNA delivery (48, 49). After this, several mRNA delivery methods that are being utilized in preclinical and clinical studies are explicated and related pros and cons are also discussed.

### Naked mRNA Delivery

The naked mRNA spontaneous uptake by cells is not satisfied, even though the transient expression was demonstrated in some studies (10, 50, 51). Some clinical trials using intratumoral injection of naked mRNA to encode tumor-associated antigens into patients with advanced melanoma and with liver cancer are currently being undertaken (43). Naked mRNAs can be passively transferred into cells of interest by electroporation, which is used on a certain type of cells in preclinical studies. When cells are treated with short high-field electric pulses, the difference of voltage along the cell membrane can cause temporary perforation, allowing mRNA molecules passively pass through the hole and spread into the cell. The electroporation-mediated delivery efficiency can be influenced by the electrical field, ionic strength of the medium, the cell type and other membrane permeability-related factors (22). Therefore, the electroporation conditions must be carefully balanced between the high uptake of synthetic mRNA and the high percentage of healthy cells. The earliest successful electroporation mRNA transfections were conducted on human hematopoietic cells and dendritic cells, which showed superior to spontaneous uptake and lipofection (48, 52). Later on, this method was also applied in other types of cells, such as stem cells and lymphocytes (53, 54). More recently, the high throughput electroporators were also successfully used to electroporate large volumes of cells (55, 56). Theoretically, electroporation and electroporator can be used in a variety of cells without special reagents, but expensive equipment is needed and more cells and mRNAs are required for each transfection. Because of the relatively high cell mortality rate, the electroporation conditions for each cell type need to be optimized to achieve the best results.

### Lipoplex- and Polyplex-Mediated Transfection

Another method for synthetic mRNA delivery is the complexation of mRNA nucleic acids with cationic lipids or polymers forming lipoplexes and polyplexes through spontaneous charge interactions (57). The carrier materials bind nucleic acids and protect their cargo from degradation by forming tight particles containing PH-sensitive molecules that escape from the endosome and enter the cytoplasm after endocytosis (22, 58). Furthermore, these carriers can be functionalized for specific cell or tissue delivery of synthetic mRNA by modifying carrier's formulation (59). Now, lipoplex- and polyplex-mediated mRNA transfection is being commonly used in various preclinical studies. However, because cationic liposomes have relatively high cytotoxicity and may interfere with cell metabolism in different cells, cationic lipid-related carriers have been mainly limited to *in vitro* studies. In recent years, more complex mRNA vectors have been created, such as PH-reactive polymer nanoparticles, which can also be systematically delivered *in vivo* (60).

### Inorganic Nanoparticle-Mediated Delivery

Although current mRNA delivery technologies are mainly concentrated on cationic polymers and liposomes, inorganic nanoparticles have also been developed. In 2009, Zohra et al. for the first time introduced that carbonate apatite inorganic nanoparticles bond with cationic liposomes of DOTAP (N-[1-(2,3-Dioleoloxy) propyl]-N, N, N-trimethyl ammonium chloride) could successfully generate high transfection efficiency of luciferase mRNA in both mitotic and non-mitotic cells (61). As an additional advantage, inorganic carbonate apatite combining with DOTAP could facilitate DOTAP-mediated mRNA expression (62). So, inorganic nanoparticle holds a promising potential to be widely used for synthetic mRNA delivery.

### Polypeptide-Mediated Delivery

As another type of synthetic vehicles, precisely designed polypeptides are also used to deliver mRNA to the cell cytoplasm (63). Amphiphilic cationic feature of the polypeptide mainly determines the mRNA delivery function. As shown in Mastrobattista et al.'s recent study, the GALA peptide functionalized the target mRNA polyplexes (PPx-GALA) in dendritic cells (DCs), and the cellular uptake of mRNA that PPx-GALA complex is 18 times higher than lipofectamine without causing cytotoxicity (64). The conjugation of precisely designed peptide to mRNA polyplexes not only promotes the mRNA expression but also plays a significant role in targeting the specific type of cells or tissue.

### Virus-Mediated Delivery

Synthetic mRNA can also be delivered into cells of interest by viral particles, which is different from conventional transfection. Such gene delivery-related viral infection requires cloning the target gene into a specific virus system and packaging specific cells to obtain the “modified” virus. Alphavirus, Sendai virus, and retrovirus have been utilized for mRNA delivery (65, 66). Retrovirus-mediated mRNA transfection can be delivered to the cytoplasm as a direct translation template for interest proteins, but the vector needs to be altered to prevent reverse translation (22). The advantage is that the infection efficiency is particularly high, especially some of the primary cells and living cells which are difficult to transfect. In addition, mRNA can avoid being degraded outside the cell by retroviral particle and the viral envelope may be modified for synthetic mRNA-transfer (67). Generally speaking, the virus system is time-consuming, expensive and complex, and may be potentially dangerous if it is improperly operated.

### Cell- Mediated Delivery

**Figure 2:**
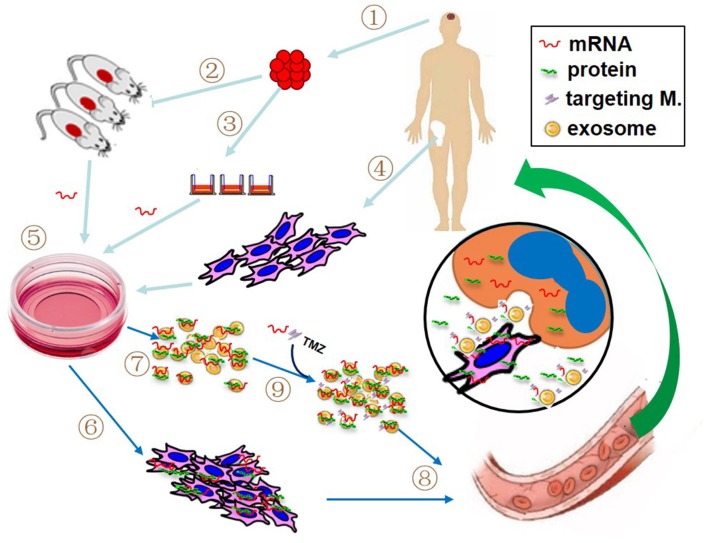
Proposed patient-tailored glioblastoma treatment with mRNA-based therapeutic modality. If practical, the target mRNA is predetermined using resected tumor tissue (1) with patient-derived xenografted animal model (2) or real-time detection (3). Meanwhile, patient's own vehicle cells (like bone marrow-derived mesenchymal stem cells, MSCs) are prepared (4). The synthetic target mRNA is delivered into cultured vehicle cells by preferred transfection method (5) and the target mRNA-bearing cells (6) and associated exosomes (7) are collected for administration (8). If necessary, additional mRNA, specific targeting molecules and/or chemotherapeutics such as temozolomide (TMZ) can be directly loaded into the isolated exosomes prior to the administration (9). The insert picture illustrates the possible action modes of mRNA in the tumor site, including mRNA product (i.e., protein) from both vehicle cell and recipient cell, cell-to-cell contact, and exosome-mediated attack molecules.

Cell-mediated gene therapy has been extensively studied for decades. A variety of types of cells have been involved in this field, including hematopoietic stem cell (HSC) (68), mesenchymal stem cell (MSC) (69), dendritic cell (DC) (70), macrophage (71), natural killer (NK) cell (72), and chimeric antigen receptor T (CAR-T) cell (73). Anti-CD19 CAR-T cell products, the first FDA approved gene therapy for patients with pre-B cell acute lymphoblastic leukemia or B-cell lymphomas, have revolutionized anti-cancer therapy, giving a new treatment option for patients who have difficulty to receive standard treatment (74). DNA-based engineering strategies have been used in the majority of cell-mediated gene therapies. However, the advantage of mRNA transfection is that the rate and duration of target gene expression can be well-managed by adjusting the quantity of mRNA, which may avoid some DNA-engineered cell-induced adverse events, such as cytokine release syndrome following the infusion of CAR-expressing T cells. Recently, as an alternative modality, mRNA has been utilized for cell-mediated gene delivery. The studied cell type includes DC (75), NK cell (72), T cell (76), HSC (77), and MSC (16, 78, 79). The most notable advantage of using cell-mediated mRNA delivery lies in vehicle cell's homing capacity. The target mRNAs can be transfected into vehicle cells by preferred delivery method, such as electroporation, viral or non-viral delivery. As illustrated in Figure 2, at the target site, mRNA-carried message can be transferred to recipient cells through several pathways: (1) cell-to-cell contact, such as DCs and CAR-T cell; (2) encoded protein produced in the vehicle cells, in the case of secreting protein or proteins containing transacting activator of transcription (TAT) segment; (3) parent cell-derived exosomes containing both mRNA and protein. Several clinical trials have been reported to use autologous DCs loaded with mRNA as a treatment of various cancers (22). In our recent work, the therapeutic efficacy of anticancer gene-engineered MSCs was also demonstrated in glioblastoma animal model (16).

### Exosome-Mediated Delivery

As the key mediators for intercellular communications, exosomes play an important part in mRNA delivery. Exosomes are nanoscale membrane vesicles mainly composed of circular double-layer lipid membranes and intracapsular contents (80). During the biogenesis of exosomes, the vesicular membrane is formed through two steps of reverse invagination of the cellular plasma membrane, resulting in vesical membrane outside-facing-out. The biological significance of this property is 2-fold. Firstly, this membrane orientation is a necessary prerequisite for the application of exosomes to targeted cancer therapy due to the targeting molecules from mother cells are also exist in the exosomes (81). Secondly, the content of vesicles is closely connected with plasma membrane reverse invagination. Theoretically, anything in the cell cytoplasm can be inwrapped in exosomes (82, 83), including synthetic mRNA from transfected parent cells. Therefore, the delivered synthetic mRNA could exist inside of exosomes in two forms, mRNA and/or relevant protein (Figure 2). Furthermore, synthetic mRNA and chemotherapeutic drugs can also be directly loaded into isolated exosomes through conventional transfection technology (84). Isolated exosomes can be passively transmitted throughout the body, but their ability to target distribution depends primarily on the surface-derived targeting molecules form parent cells (84). In the recipient cells, exosomes are primarily absorbed into the cell by endocytosis, membrane fusion, or receptor-mediated internalization (85). Due to the cell-free nature and biological characteristics, exosomes replace cell-based modality (like CAR-T) and directly attack tumor cells. In regards to cancer therapy, exosomes have following advantages. First, exosomes can be used as “off-the-shelf” reagents so that the doses can be controlled according to the therapeutic condition. Second, their nanoscale size provides the possibility for solid tumor therapy such as GBM. Third, the combined and/or alternate use of cell-based and cell-free platforms will enhance the application value for cell-based cancer therapy (86).

## The Therapeutic Prospects of mRNA-based Gene Therapy for Glioblastoma

The reason why GBM is difficult to treat rests with its special biological characteristics, such as located in the brain and defended by both BBB and BTB, high heterogeneity, diffusing invasiveness, and capacity to resist conventional therapies. As mechanical barriers, BBB and BTB protect GBM from certain therapeutic agents. The high heterogeneity of GBM probably means that it cannot be well-cured by any single particular drug, especially biomarker-related agents. Its invasive growth property determines the highest recurrence rate after surgical resection, and its high capacity of drug resistance suggests that it cannot be efficiently treated by the sustained use of any specific drug. Taken together, an ideal anti-GBM strategy should adapt all challenges and meet the following requirements: (1) Anticancer actions are specifically confined to the tumor site; (2) personalized treatment plan according to the sensitivity of anticancer drugs predetermined; (3) multiple anticancer mechanisms can work simultaneously; and (4) the anticancer agents can be adaptively replaced. Of course, because GBM grows in a restricted intracranial environment, if practical, surgical resection is always the first treatment upon diagnosis, otherwise, the patients may die from some GBM-related complications, such as cerebral hernia, and we may not have the opportunity to provide any therapeutic intervention.

### The Use of mRNA-Based Anticancer Gene Products as the Ultimate Attackers of the Tumor Has Special Superiority for GBM Treatment

As described earlier, nano-scaled target mRNA-bearing lipoplexes, polyplexes, liposomes and exosomes are all able to cross BBB and BTB in the brain (87, 88), overcoming the natural barriers. The rapid and transient expression nature of synthetic mRNA, which has been considered the biggest weakness of using mRNA for gene therapy, is just suitable for dealing with GBM's complexity and variability through its adaptive convertibility. It is also because of its short-term high-level expression, mRNA-based gene therapy has been intensively and almost exclusively focused on cancer treatment (89, 90). The selection of anti-oncogene depends mainly on the specific situation of each patient. If possible, predetermination should be performed using *in vitro* real-time detection of tumor cells or patient-derived xenografted animal model immediately after surgical resection (91, 92).

### The Highly Specific Targeting Capability of mRNA-Based Modality Is Essential to Achieve the Most Effective Treatment of Cancer With Limited Side Effects

In this mRNA-based therapeutic modality, the GBM targeting can be achieved at least in three levels. (1) Cell level. The tumor-homing property has been verified in several types of cells, including NK cells, DCs, and MSCs (72, 93, 94). In addition to their ability of tumor-directed migration and incorporation, these types of cells are easy acquisition and fast *ex vivo* expansion. More importantly, they are feasible for autologous transplantation. These cells are also able to cross BBB especially under brain tumorous condition (95, 96). The targeting capacity of these cells can be further enhanced by transfecting specific targeting molecules (e.g., CAR-T). The therapeutic efficacy of GBM by cell-mediated and mRNA-based modality has heterogeneity in preclinical studies and a number of them are currently in different phases of clinical trials (16, 20, 96). (2) Exosome level. As aforementioned, anticancer mRNA-bearing exosomes are able to target tumor cells directed by the targeting molecules in their membrane, which originate from their parent cells. Furthermore, additional targeting molecules can be loaded into exosomes after their isolation. Several GBM-specific targeting peptides have been precisely investigated. These peptides act on a different part of the tumor cell through different mechanisms (97). (3) Molecular-level. Under certain circumstances, the ultimate attack molecules kill tumor cells in a tumor-specific manner. For example, tumor necrosis factor-related apoptosis inducing ligand (TRAIL) induces apoptosis specifically in tumor cells through binding with death receptors. This target specificity is determined by the differential expression of death receptors in tumor cells (98).

### The Administration Route of mRNA Is a Patient-Tailored Selection

The selection of delivery routes of synthetic mRNA to GBM patients depends on the general evaluation of various parameters, including patient's glioma grade, stage, surgical, and chemotherapy history, as well as the available forms of synthetic mRNA carriers. In general, intravenous infusion is the safest and most practical administration method for various forms of synthetic mRNA. Other options include local injection, cerebrospinal fluid infusion, interventional infusion and administration through the nasopharyngeal pathway. However, it is worth noting that the alternate use and sometimes a combination of different mRNA content and different delivery method could be significantly beneficial for the patients with GBM. In our previous clinical trial, the combination of local application of MSCs during surgical operation and intravenous infusion of MSCs after surgery achieved an ideal outcome (99).

Although no clinical trials of mRNA-based GBM therapy have yet been completed, some are underway. A phase II randomized, blinded trial of CMV RNA-pulsed dendritic cells with tetanus-diphtheria toxoid vaccine in patients with newly-diagnosed glioblastoma is ongoing^1^. The mRNA-based therapy has not been widely adopted in treating GBM, but it had made great progress in other diseases. A phase 1 clinical trial showed that rabies vaccination based on mRNA encoding was safe and could produce functional antibodies (100). Martin Sebastian et al. reported a phase I/II a study of mRNA-based cancer immunotherapy on non-small cell lung cancer. The patients of this trial were well-tolerated and the antigen-specific immune responses were detected in 63% of assessable patients after five injections (101). The therapeutic prospects of mRNA-based gene therapy for GBM could proliferate when noted obstacles are overcome, such as the targeted delivery *in vivo*, rapid degradation, and short half-life. We also have to address the repetitive application and sufficient delivery efficiency in the brain.

## Conclusions

The dissatisfactory outcome of GBM treatment has retained for decades. The reason why GBM is difficult to treat rests with its special biological characteristics such as high heterogeneity, diffusing invasiveness, and capacity to resist conventional therapies, as well as the existence of biological barriers, e.g., BBB and BTB. Compared with conventional DNA-based strategy, synthetic mRNA-mediated therapeutic modality holds many superior features including easy manipulation, rapid and transient expression, and adaptive convertibility without mutagenesis, which is suitable for dealing with glioblastoma's complexity and variability. Synthetic anticancer mRNAs carried by various vehicles, such as organic or inorganic nanoparticle-encapsulated complexes, patient-derived DCs or MSCs and their corresponding exosomes, act as the ultimate attackers of the tumor across biological barriers. In this mRNA-based therapeutic modality, specifically targeted GBM treatment can be guaranteed by adding targeting molecules at certain levels. The choice of mRNA-bearing vehicle and administration method is a fully patient-tailored selection. To date, mRNA has been used in preclinical clinical trials such as cancer immunotherapy, infectious disease control and regenerative medicine (24, 102, 103), but, anticancer treatment is the most developed application for mRNA (89, 104). The mRNA-based therapeutic modality holds a great promising potential to be efficiently utilized for the patients with GBM.

## Ethics Statement

Ethical approval was waived since we used only publicly available data and materials in this study.

## Author Contributions

XT, LZ, and QC designed this research. RF, KH, and HP collected relevant literatures. XT and LD wrote and revised the manuscript. SZ helped to correct the language. All authors have read and approved the final manuscript.

### Conflict of Interest

The authors declare that the research was conducted in the absence of any commercial or financial relationships that could be construed as a potential conflict of interest.

## Footnotes

^1^www.clinicaltrials.gov

## Abbreviations

mRNA: messenger Ribonucleic Acid
GBM: Glioblastoma multiforme
WHO: World Health Organization
BBB: blood-brain barrier
BTB: brain-tumor barrier
pDNA: DNA-based plasmid
ARCA: anti-reverse cap analogs
PCR: Polymerase Chain Reaction
DOTAP: N-[1-(2, 3-dioleoloxy)propyl]-N,N,N-trimethyl ammonium chloride
DCs: dendritic cells
HSC: hematopoietic stem cell
MSC: mesenchymal stem cell
NK: natural killer
CAR-T: chimeric antigen receptor T
TRAIL: tumor necrosis factor-related apoptosis inducing ligand.
